# Determination of permeability data and 3-D modelling of the host rock and sinters from a geothermal field: Los Geysers, northern Trans-Mexican Volcanic Field

**DOI:** 10.1016/j.dib.2022.108637

**Published:** 2022-09-24

**Authors:** Mohamed Ali Elabd, Reneé González-Guzmán, Bodo Weber, Corina Solís, Rubén Bernard-Romero, Fernando Velasco-Tapia, Pedro Marín-Camacho

**Affiliations:** a Department of Geology, Faculty of Science, Aswan University, Sahary City, Airport Road, Aswan 81528, Egypt; b Centro de Geociencias, Universidad Nacional Autónoma de México, Campus Juriquilla, Juriquilla, Querétaro 76230, Mexico; c Departamento de Geología, División de Ciencias de la Tierra, CICESE, Carretera Ensenada-Tijuana 3918, Zona Playitas, Ensenada, Baja California 22860, Mexico; d Instituto de Física, Laboratorio de Espectrometría de Masas con Acelerador (LEMA), Circuito de la Investigación Científica, Universidad Nacional Autónoma de México, CP. 04510, Ciudad Universitaria, Ciudad de México, Mexico; e Facultad de Ciencias de la Tierra, Universidad Autónoma de Nuevo León, Carretera Linares-Cerro Prieto km 8, Linares, Nuevo León 67700, Mexico

**Keywords:** Digital image analysis, Digital rock physics, n-XRT computed tomography, Volcanic rocks, Hydrothermal deposits, Petrophysics

## Abstract

This data article describes the connected pore cluster data from segmented nano-images of rocks related to a geothermal system. The collected samples include two (2) vesicle-amygdaloidal basalt (host rock) and four (4) horizons collected from a siliceous sinter mound (rock precipitated from hot waters). All the samples have undergone computed tomography scanning using a SkyScan 2211 multiscale X-ray nano-CT system (Bruker®), and the slices were analyzed using the Digital Rock Physics (DRP) approach. Pore volume and fluid permeability in the three directions were calculated with scripts of Python (v.3.9) and the visualizations of the 3D models were run with Paraview (v.5.10) software. The petrophysical properties, diagrams, and figures were produced by stacking the 2D projections (8-bit grayscale *.png images format) from the scanning. Raw data (images) were deposited in a repository, which has granted a persistent identifier (Mendeley Data: https://data.mendeley.com/datasets/srpxhpd37p/2). This article provides a study case to handle the data that test the interconnectivity and ability to transport fluids and/or exogenous matter carried during high-flow events in rocks outcropping at the surface level of a geothermal system.

**Specifications Table**

**Table:** 

Subject	Earth Science, Materials Science
Specific subject area	Petrophysical features of rocks
Type of data	(a) Grayscale images (b) Table (c) Graph (d) Figure
How the data were acquired	(a) The raw data (8-bit *.png format) was acquired through computed tomography scanning using a SkyScan 2211 multiscale X-ray nano-CT system (Bruker microCT®). (b) The images were stacked, reconstructed and handled by SNOW and OpenPNM algorithms of Python (v.3.9). The visualization was conducted using ParaView (v.5.10) software.
Data format	(a) Raw: n-XRT computed tomography image slices (*.png). (b) True metadata: text files (*.txt) containing information about the pixel size (um) in the tomography raw-slices, characteristic of the source, i.e. source voltage (kV), source current (μA), source type, and other acquisition parameters. (c) Processed: segmented CT images (d) Analyzed: text file, graphs, and figures (*.docx, *.xlsx, *.vtk, and *.png)
Description of data collection	The general settings for the n-XRT analyses of the rock samples were as follows: Volume of the samples: ∽100–200 mm^3^ Source voltage: 110 kV Target current: 80–90 μA Source target type: Tungsten (W) Exposure time: 100–150 ms Image Pixel Size (μm): 10.00 avg. Projections: 1042 Reconstruction angular range: 208.40 (deg)
Data source location	• Institution: Centro de Investigación Científica y de Educación Superior de Ensenada, Baja California (CICESE) • City: Ensenada, Baja California • Country: Mexico • Location of the collected samples: 20.53624°; −100.54657°; ∼1800 m.a.s.l. (Los Geysers geothermal field, Mexico)
Data accessibility	Repository name: Mendeley Data Data identification number: doi:10.17632/srpxhpd37p.2 Direct URL to data: https://data.mendeley.com/datasets/srpxhpd37p/2
Related research article	Reneé González-Guzmán, Bodo Weber, Mohamed Ali Elabd, Corina Solís, Rubén Bernard-Romero, Fernando Velasco-Tapia, Pedro Marín-Camacho, 2022. Petrogenesis of Holocene siliceous sinters from the Los Geysers geothermal field, northern Trans-Mexican Volcanic Belt, Journal of Volcanology and Geothermal Research, 431, 107640, https://doi.org/10.1016/j.jvolgeores.2022.107640.

## Value of the Data

• The provided data is of invaluable importance as it represents non distructive 3D volumes for hydrothermal precipitates along with their host rock where both show the internal structure. Its importance also exist in being a representative for similar deposits around the world or share similar physico-chemical conditions of similar system, as reveald by González-Guzmán et al. (2022).
• Petrophysicits and geologists are the main researchers who can reuse these data.
• The provided 3D nano-CT data can be used for petrophysical comparative studies on similar hydrothermal precipitations/ systems.
• The 2-phase flow of the hydrothermal fluid along with its contained gas can be modelled for environmental history purposes.
• The provided data for the host rock can be used for studying the cementation processes by modelling the filling of pore by silicious precipitations through what is called the process-based rock modelling.

## 1 Data Description

The raw data presented here is available as a Mendeley data set [1]. It contains 998 grayscale image slices (*.png) per sample, and one *.csv file which describes in detail the parameters used during the scanning for each sample:

1. ARU-144 – Vesicle-amygdaloidal basalt collected at the center of the geothermal field.
2. ARU-145 – Vesicle-amygdaloidal basalt collected at the southwestern corner of the geothermal field.
3. CIC7a – layer 1 (bottom) of a relict sinter mound from Los Geysers geothermal field.
4. CIC7b – layer 2 of a relict sinter mound from Los Geysers geothermal field.
5. CIC7c – layer 3 of a relict sinter mound from Los Geysers geothermal field.
6. CIC7d – layer 4 (top) of a relict sinter mound from Los Geysers geothermal field.

The processed experimental data and their construction strategy are fully detailed in the next section. Table 1 presents the calculated coefficient of **permeability (Darcy) of the six samples**. Fig. 2 shows the two-point correlation function of the six samples. The exported images along with their extracted pore network are shown in Fig. 3, Fig. 4, Fig. 5, Fig. 6, Fig. 7, Fig. 8, Fig. 9, Fig. 10. Moreover, the Mercury Intrusion Capillary Pressure (MICP; Fig. 11), was simulated on the six samples using the percolation algorithm in the OpenPNM python package [2]. The Hg saturation is relative to the sample's porosity meaning the Hg saturation in Fig. 11 is normalized between 0-1, regardless of the Hg intruded quantity. The MICP values of both ARU-144 and ARU-145 represent only the Hg volume intruded into the surficial pore spaces on the sample's six sides.

## 2 Experimental Design, Materials and Methods

**Fig. 1:**
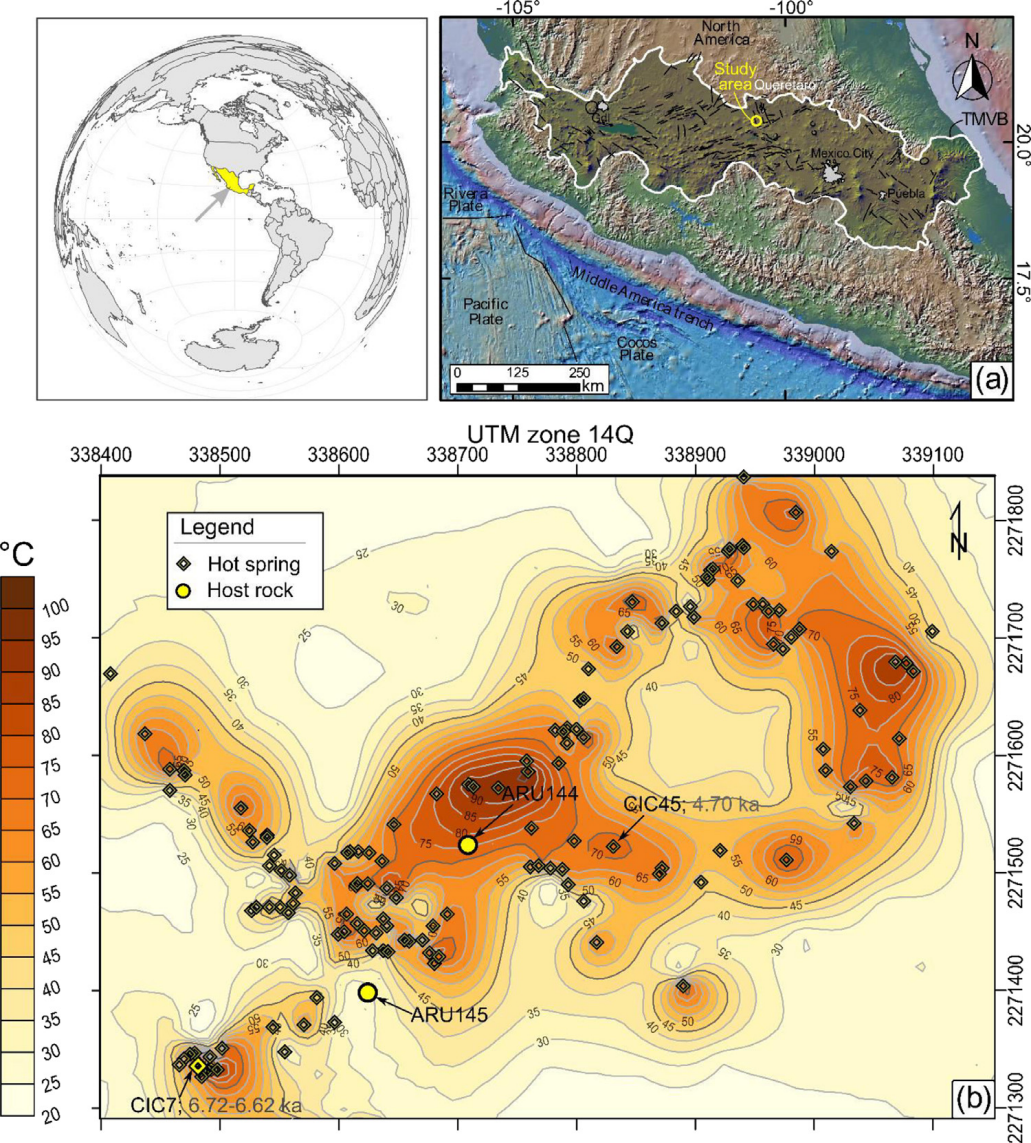
(a) General map of central Mexico showing the location of the Trans-Mexican Volcanic Belt (TMVB). (b) GIS-based map showing the area under study, temperature domains based on thermal fluid discharges, and sampling point locations [4,5]. Isotherms are drawn at intervals of 5 °C.

Six samples were collected from Los Geysers geothermal field (20.53624°, -100.54657°; ∼1800 m.a.s.l.; Fig. 1) and scanned using n-XRT computed tomography, in order to test their interconnectivity and their ability to transport fluids and/or organic matter. The analyzed samples include two aliquots collected from the local host rock and four others collected from a sinter sample. The sinter specimen corresponds to the wall of a relict sinter mound. From the whole mass, four subsamples were taken and target individual horizons. These subsamples are representative of the geothermal field. They were labeled alphabetically in the same order (from a[bottom] to d[top]). Carbonaceous material present in the pores of samples CIC7a and CIC7d was extracted for radiocarbon dating [3]. The bulk organic material extracted from the sample CIC7a yielded an age range between 6776 and 6673 cal yr B.P. (1σ), whereas the organic matter from the sample CIC7d has an age between 6674 and 6561 cal yr. B.P. (1σ). A volume about 100–200 mm^3^ of the samples were scanned using a voltage of 110 kV, target current of 80–90 μA (Tungsten as source target), and exposure time of 100–150 ms. The procedure was done for 180° with a rotation step of ∼0.2°. The resolution is controlled by sample position between the X-Ray tube (with submicron spot size) and the detector. Three-D reconstruction from a set of CT raw images employs several computational technologies to tackle the inverse problem going from 2D images to 3D visualizations and models. Here, diverse aspects of the modelling protocol and its application for calculating the poro-perm properties of the analyzed rocks is presented. All the samples have undergone CT scanning using the SkyScan 2211 multiscale X-ray nano-CT system, then analyzed using the Digital Rock Physics (DRP) approach to test their permeability, effective porosity, and capillary pressure.

DRP concerns modelling and computing the rock's physical and petrophysical properties through, mostly 2-phases (voids and solids), segmented 3D CT images of that rock by using an adequate algorithm or solver [6]. DRP workflow begins with 3D CT image acquisition, image processing, and finally computing and simulating the desired physical properties. The DRP method incorporate two categories: direct pore-scale modelling methods, where physical models or solvers, such as Lattice-Boltzmann Method (LBM) (e.g. [7,8], are used directly on the segmented image to calculate the desired properties; and the Pore Network Modelling (PNM) technique (e.g. [9], [10], [11], [12], where the pore space is simplified into pores and throats which are in turn represented by simple geometrical shapes, especially, spheres and cylinders. For an overview, the author recommends [13,14]. In this work, the PNM approach was used, as it is computationally affordable, in comparison to the direct simulation techniques that would take days to calculate these petrophysical properties. The PNM technique has two major steps: simplifying the irregular pore space into regular spheres and cylinders and this is called the pore network extraction; and calculating the rock properties. Considering the first step, the SNOW algorithm [11] which is implemented in the Porespy [15] Python package was used. This algorithm uses the distance map and watershed concepts to simplify the pore space into regular spheres and cylinders. The second step, therefore, that includes calculating the fluid permeability of rock samples in the three directions, was accomplished using the OpenPNM [2] Python package on the extracted pore network from the first step. Both the extracted pore networks and the 3D porous rock samples were then exported to be visualized in the Paraview ([16]; v.5.10) software.

**Fig. 2:**
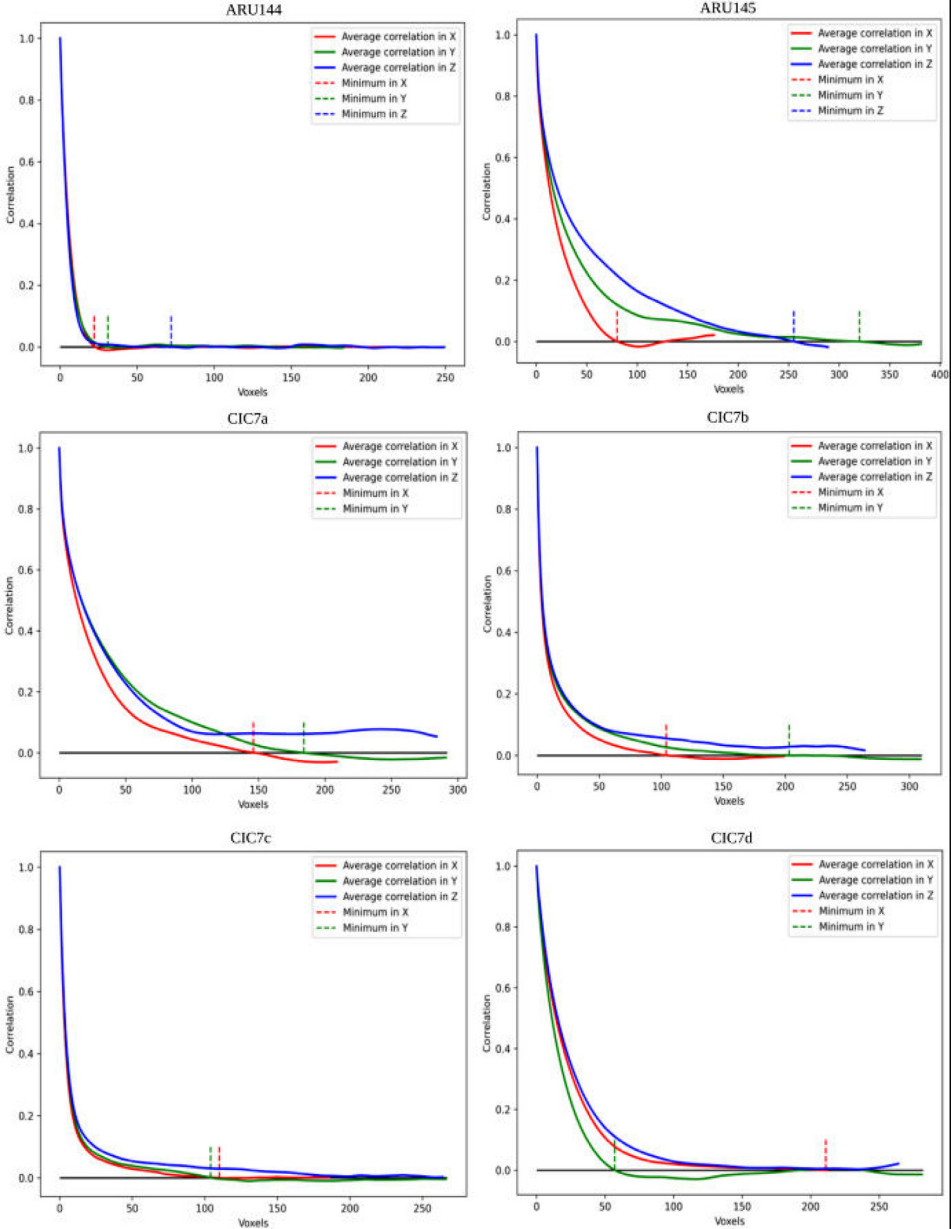
Two-point correlation function of the six samples (Voxels *vs*. Correlation).

Firstly, the six samples were segmented using the Otsu's method. The sizes and porosities of the six samples are shown in Table 1. Then, the homogeneity of the rock samples is measured using the two-point correlation function reported by [17]. This is to test the ability of the scanned sample for having a representative elementary volume (REV). Additionally, this pore-pore, two-point correlation function describes the probability of finding every possible pair of voxels, with a known distance between each other, belonging to the same phase (pore) [9]. The 2-points correlation functions of the samples are shown in Fig. 2 which reveals that ARU-144 and CIC7d samples are the only ones that may have a representative elementary volume, while the other samples are heterogenous. Consequently, all the samples were downscaled by a factor 0.5 for our workstation to be able to handle these sizes in a short time. Nonetheless, porosity-based REVs were extracted from ARU-144 and CIC7d and compared with the resized data in order to assure that the downscaling reserves the same morphology of the original images. We use Google Colab [18] cloud as our workstation for that purpose. All the samples were then undergone network extraction, permeability estimation, and finally both image datasets and their pore networks were exported to Paraview (v.5.10) for 3D visualization.

**Table 1:** Permeability of the sinters and their host.

	Permeability (Darcy) (D)		
Sample	x	y	z
ARU-144	0	0	0
ARU-145	0	0	0
CIC7a	22.6	0	34.4
CIC7b	∼0	5.3	10.6
CIC7c	1.3	2.3	9.2
CIC7d	45	2.9	16

In the PNM methodology, the pore space is classified into pore bodies, represented by spheres, and pore throats which connect 2 pore bodies and are represented by tubes. Due to the computational cost, the data was resized by a factor 0.5 then the pore network was extracted and finally the permeability was calculated. To test the reliability of the resizing method, the porosity (φTot) was tested and found to be like the original. Thus, downscaling the big image datasets into a smaller volume is a good and accepted concept as it reserves the original morphology. From this perspective the images samples can be used in full volumes with no need to subset a REV. While the porosity-based REV showed closer relation to the full data in sample ARU-144, in sample CIC7d it has missed the most important characteristic of the sample which is the fracture. That is why the REV measured permeability is approximately half of the full sample's permeability which in turn supports our choice for downscaling the data. The models are shown below:

**Sample 1: ARU-144**

**1- Original:**

Voxel side length = 14µm
Sample's total porosity (φTot): 16.48 %
Size = 859 × 740 × 1001 voxels
Size = 12 mm × 10 mm × 14 mm

**2- Using a Representative Elementary Volume (REV)**

Size = 500 × 500 × 500 voxels
Size = 7 mm × 7 mm × 7 mm
REV total porosity (φTot): 16.36 %

**Fig. 3:**
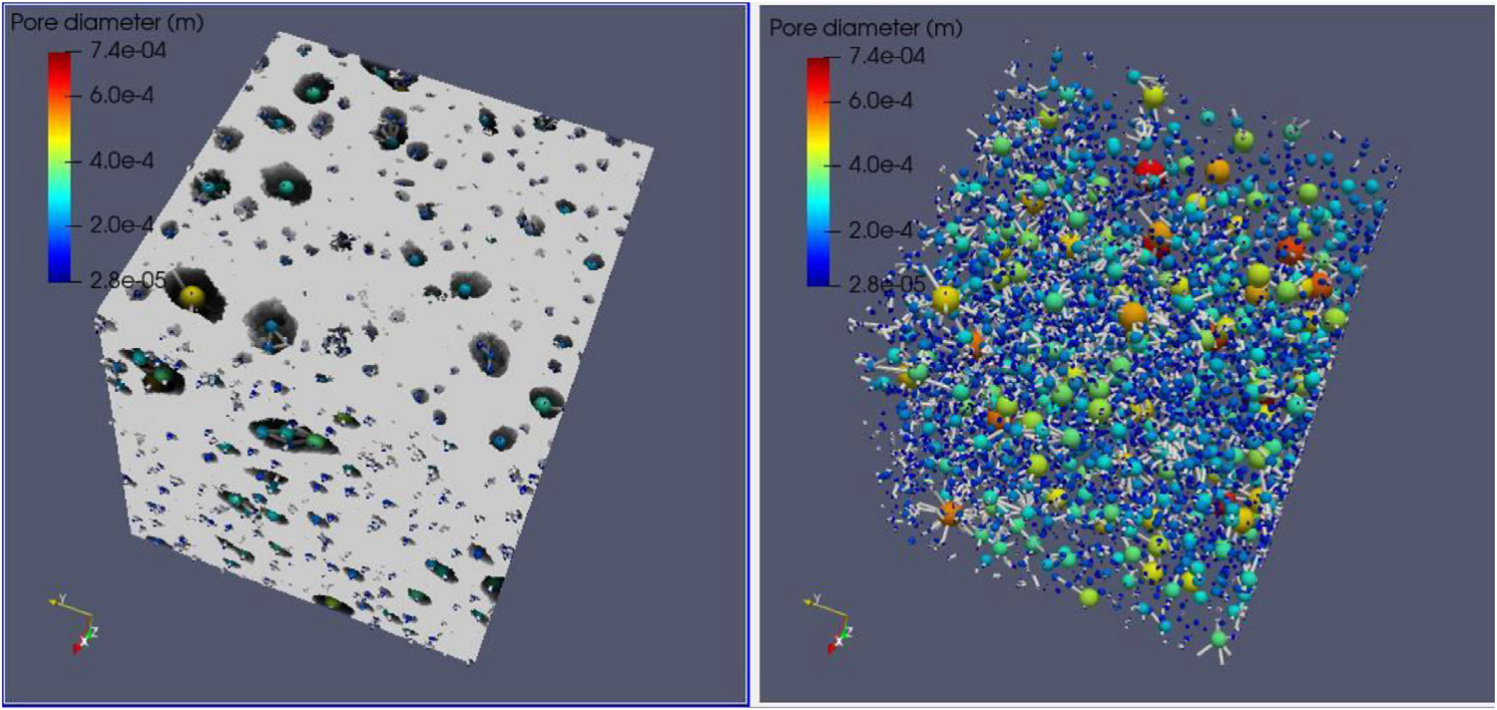
Full scanned samples along with their extracted pore network of the sample ARU-144. The sample cannot transport fluids in any direction as it is composed of separated pore clusters.

**3- Resizing Data**

Voxel side length = 28 µm
Sample total porosity (φTot): 16.48 %; Size = 430 × 370 × 500

**Fig. 4:**
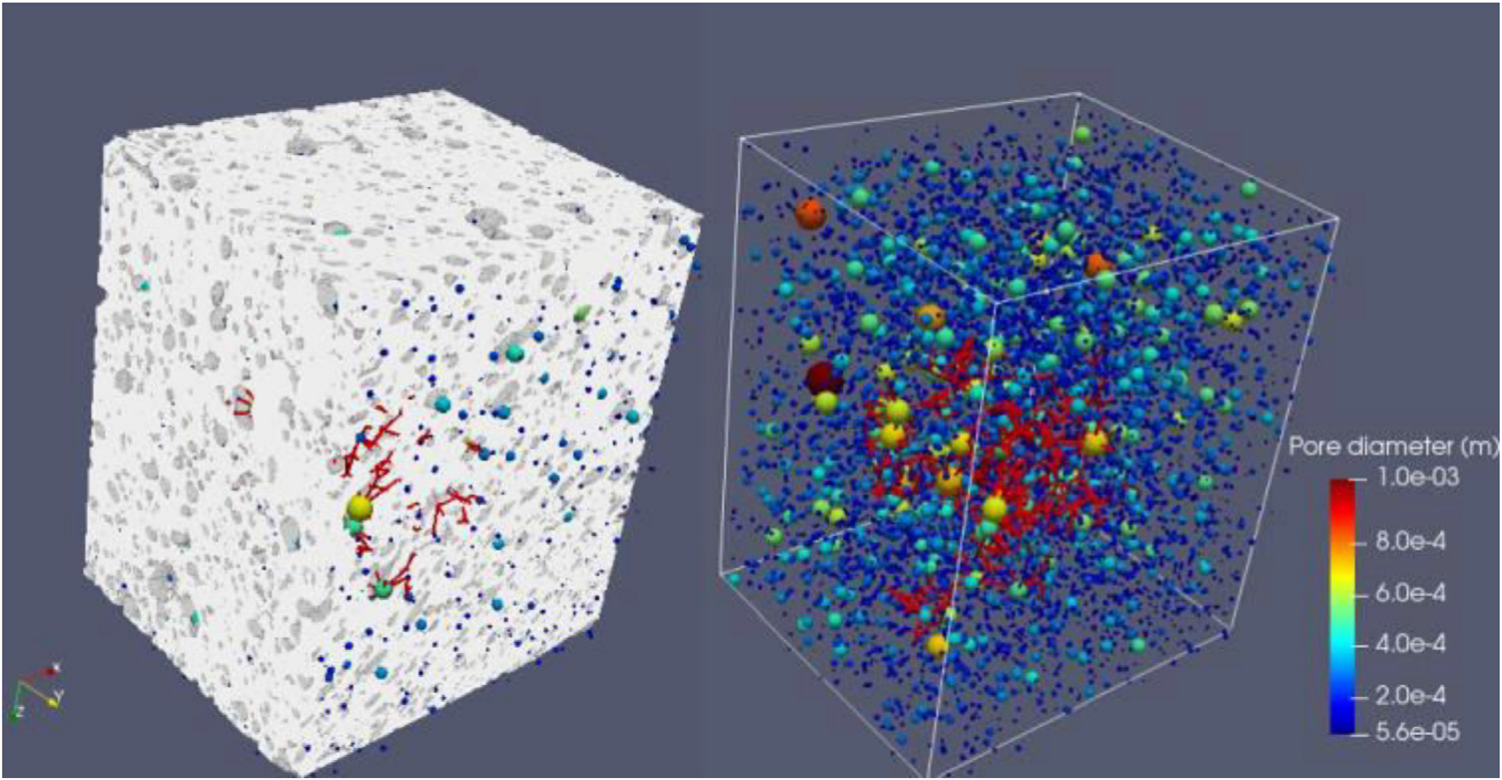
Full scanned samples along with their extracted pore network of the sample ARU-144 (Resizing data). The sample cannot transport fluids in any direction as it is composed of separated pore clusters. Red threads represent pathways of connected pore bodies.
Voxels size = 12 mm × 10 mm × 14 mm

**Sample 2: ARU-145**

**1- Original**

Pixel side length = 14.5µm
Sample total porosity (φTot): 21.7 %
Size = 710 × 1530 × 1161 voxels
Size = 10.3 mm × 22.18 mm × 16.8 mm

**2- Resized**

Pixel side length = 29 µm
Sample total porosity (φTot): 21.7 %
Size = 355 × 765 × 580 voxels
Size = 10.3 mm × 22.18 mm × 16.8 mm

**Fig. 5:**
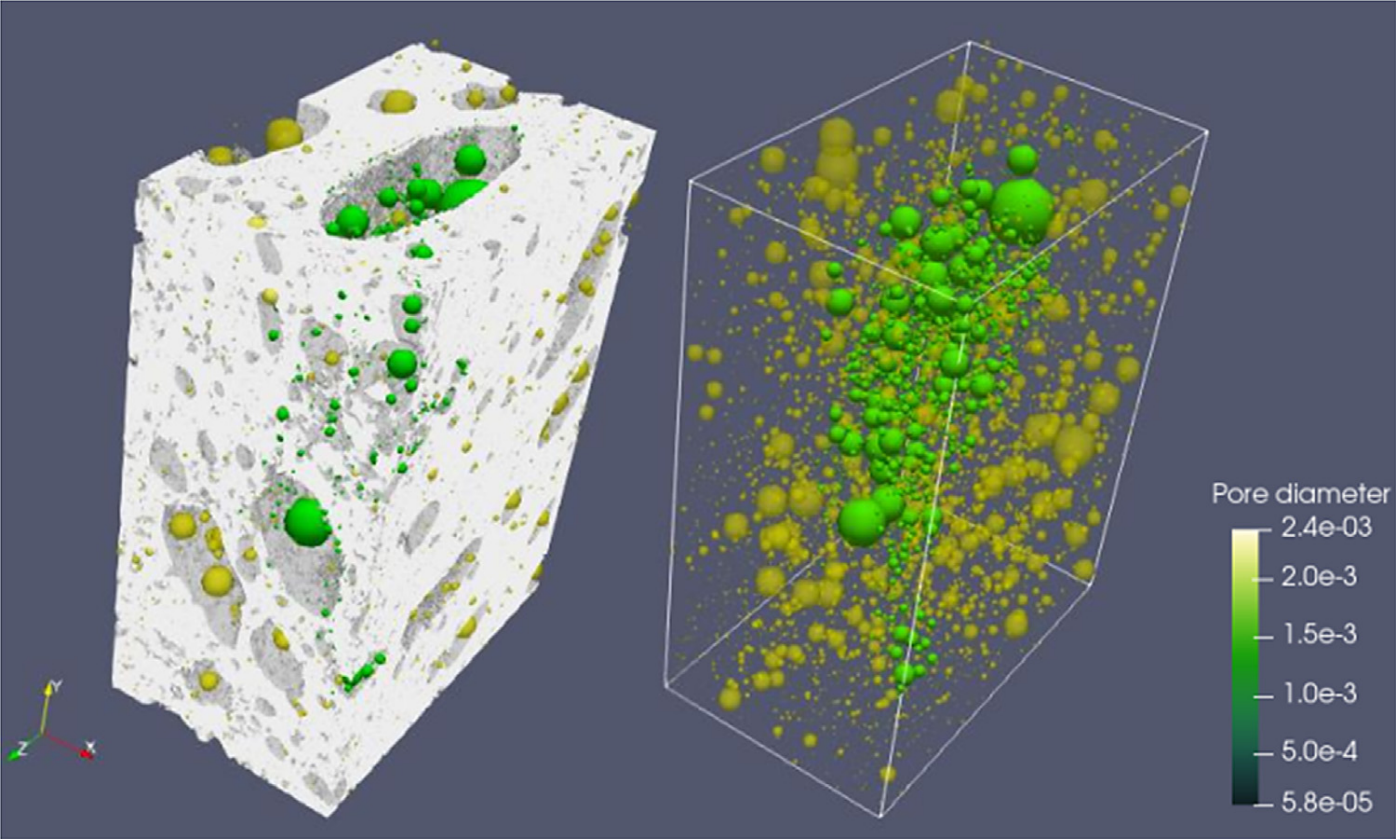
Full scanned samples along with their extracted pore network of the sample ARU-145. The sample cannot transport fluids in any direction. The green colored pore bodies are the only connected pores and the pale yellow are the isolated ones. The color bar is only for showing the widely various range of pore diameters.

**Sample 3: CIC7a**

**1- Original**

Pixel side length = 10 µm
Sample total porosity (φTot): 10.4 %
Size = 840 × 1170 × 1140 voxels
Size = 8.4 mm × 11.7 mm × 11.4 mm

**2- Resized**

Pixel side length = 20 µm
Size = 420 × 585 × 570 voxels
Size = 8.4 mm × 11.7 mm × 11.4 mm

**Fig. 6:**
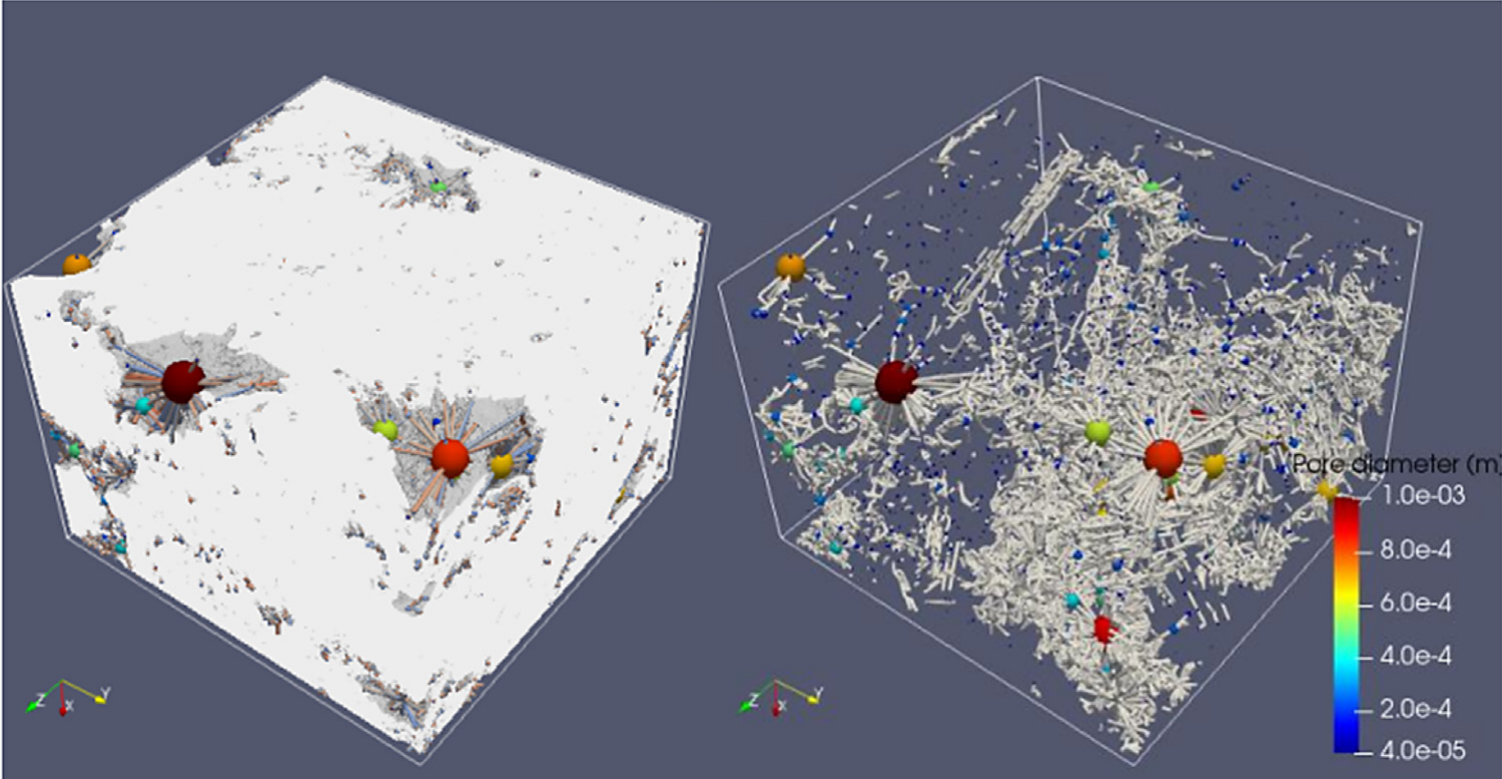
Full scanned samples along with their extracted pore network of the sample CIC7a. The sample can easily transport fluids in X and Z directions as the sample's permeability is 22.6 Darcy and 34.4 Darcy in X and Z, respectively, while it cannot transport water in the Y direction.

**Sample 4: CIC7b**

**1- Original**

Pixel side length = 13.5 µm
Sample total porosity (φTot): 19 %
Size = 800 × 1240 × 1060 voxels
Size = 10.8 mm × 16.7 mm × 14.3 mm

**2- Resized**

Pixel side length = 27 µm
Size = 400 × 620 × 530 voxels
Size = 10.8 mm × 16.7 mm × 14.3 mm

**Fig. 7:**
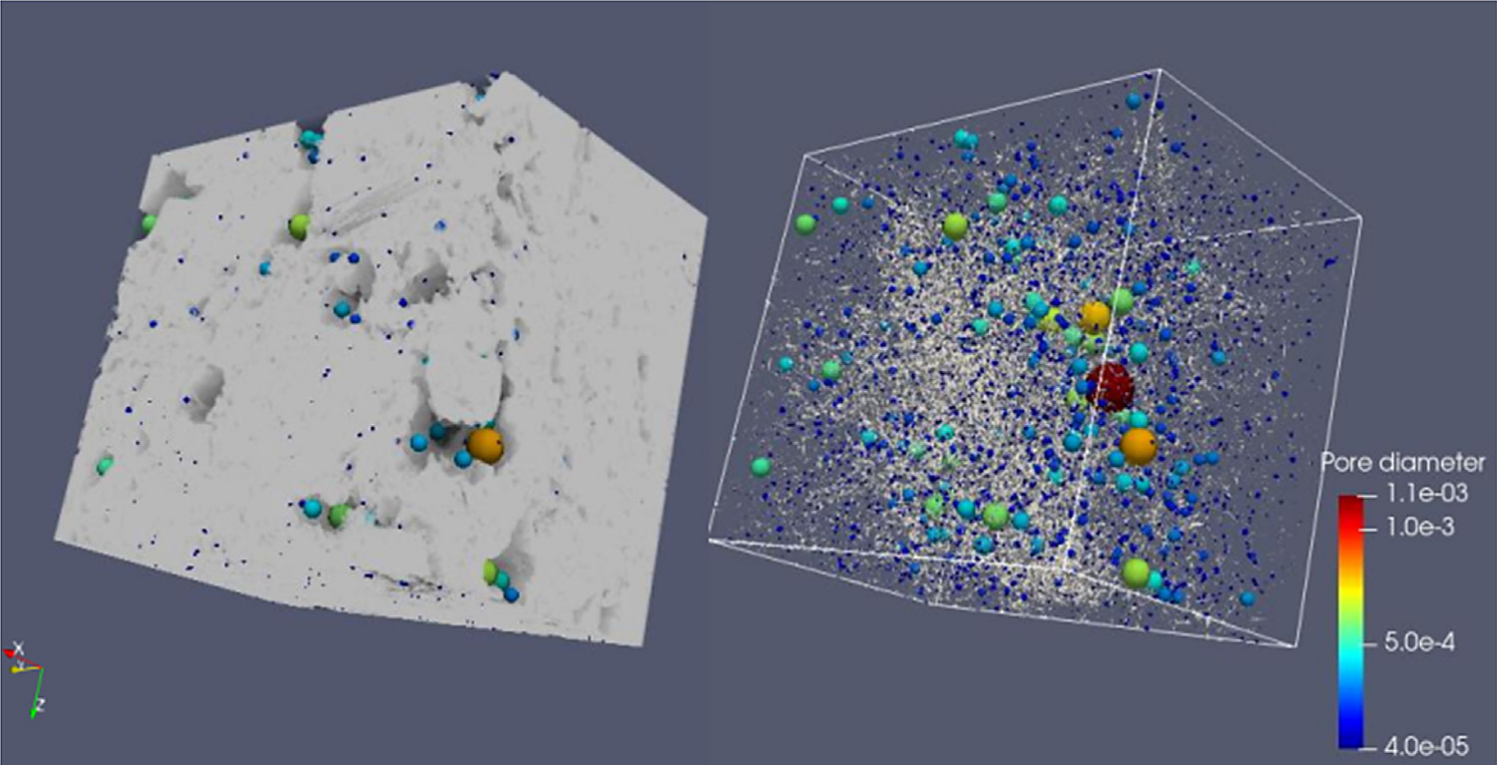
Full scanned samples along with their extracted pore network of the sample CIC7b. The sample can transport fluids in Y and Z directions and barely in X as the sample's permeability is 1.3 mD, 5.3 D and 10.6 D in X, Y and Z, respectively.

**Sample 5: CIC7c**

**1- Original**

Voxel side length = 10 µm
Sample's total porosity (φTot): 24 %
Size = 750 × 1070 × 1060 voxels
Size = 7.5 mm × 10.7 mm × 10.6 mm

**2- Resized**

Voxel side length = 20 µm
Size = 375 × 535 × 530 voxels
Size = 7.5 mm × 10.7 mm × 10.6 mm

**Fig. 8:**
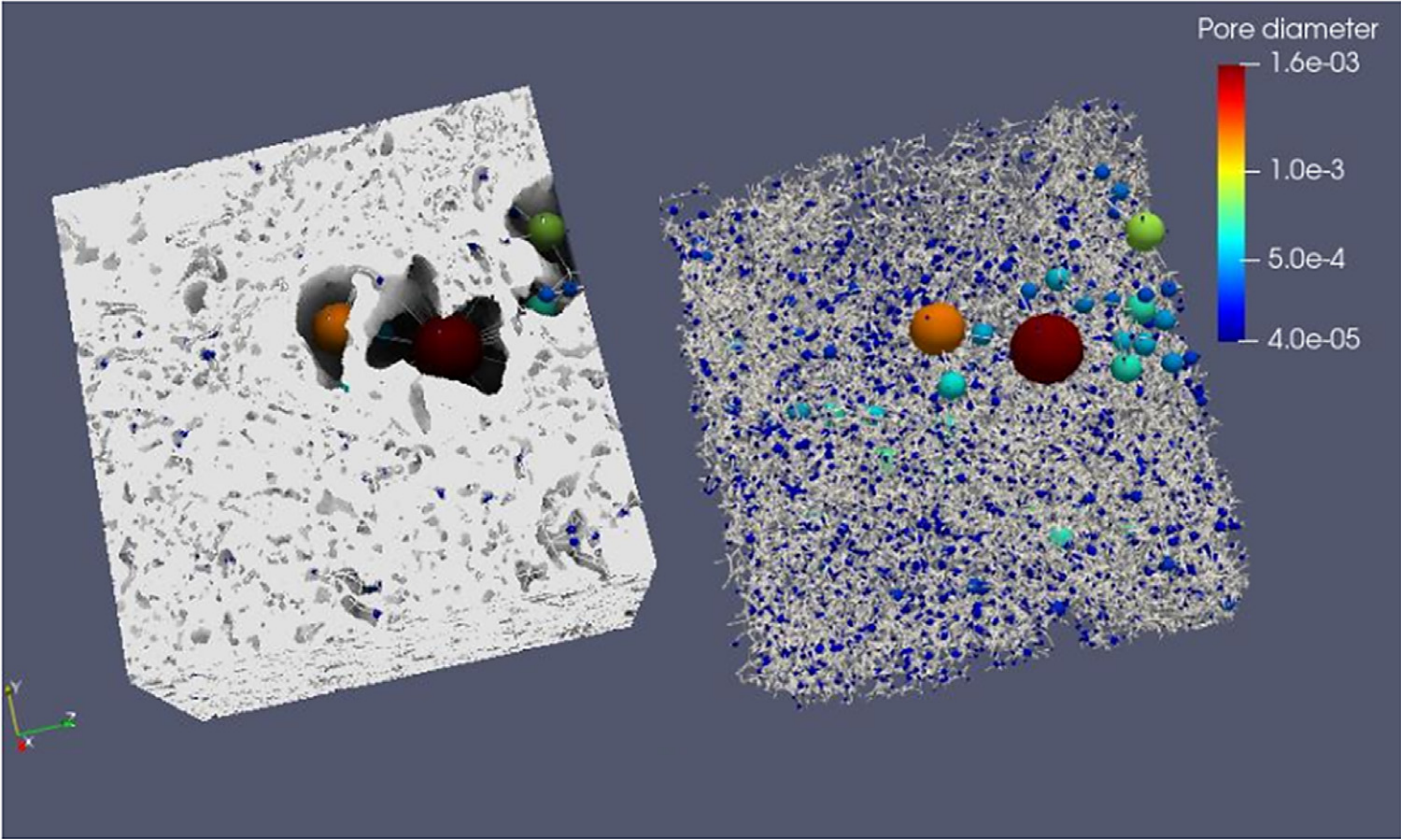
Full scanned samples along with their extracted pore network of the sample CIC7c. The sample can transport fluids in X, Y and Z directions. The sample's permeability is 1.3 D, 2.3 D and 9.2 D in X, Y and Z, respectively.

**Sample 6: CIC7d**

**1- Original**

Voxel side length = 10 µm
Sample's total porosity (φTot): 13.5 %
Size = 854 × 1228 × 1161 voxels
Size = 8.5 mm × 12.2 mm × 11.6 mm

**2- Rev**

Voxel side length = 10 µm
REV total porosity (φTot): 12.5 %
REV size = 5 mm × 5 mm × 5 mm

**Fig. 9:**
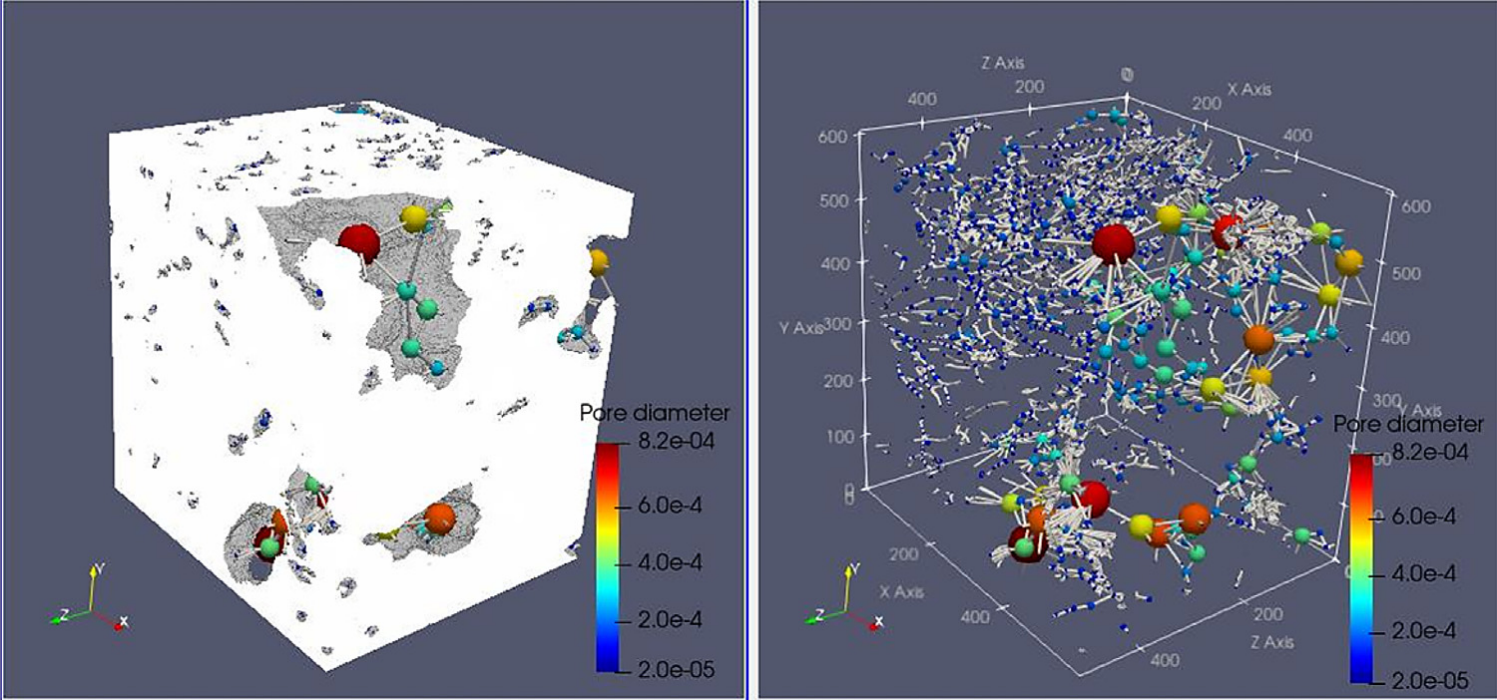
Full scanned samples along with their extracted pore network of the sample CIC7d REV.

**3- Resized Data:**

Voxel side length = 20 µm
Sample's total porosity (φTot): 13.5 %
Size = 427 × 564 × 530 voxels
Size = 4.2 mm × 5.6 mm × 5.3 mm

**Fig. 10:**
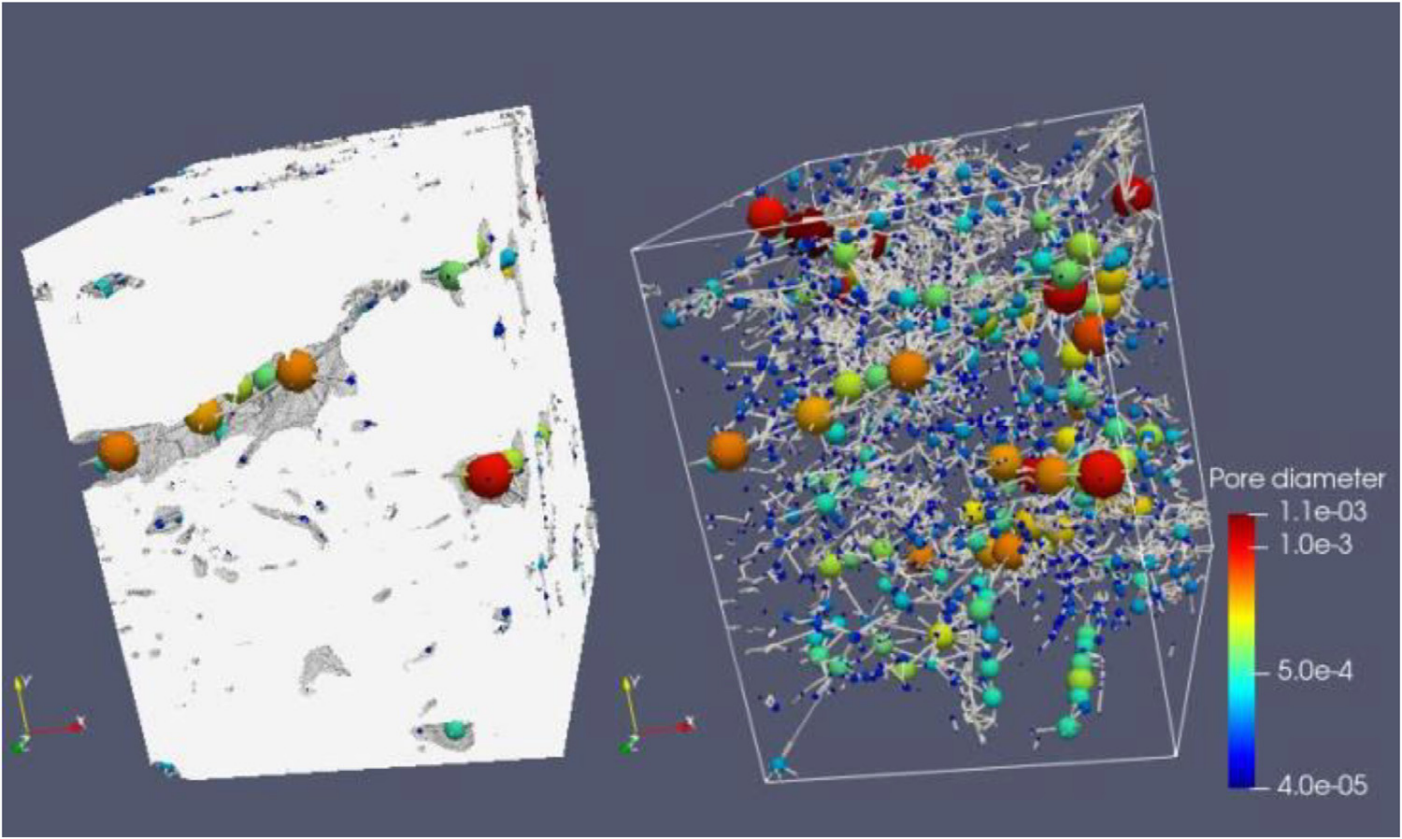
Full scanned samples along with their extracted pore network of the sample CIC7d (resizing). The sample can easily transport fluids in all directions. The sample's permeability is 45 D, 2.9 D, and 16 D in X, Y, and Z, respectively. The sample incorporates various types of pore bodies, especially, elongated/ fractures.

**Fig. 11:**
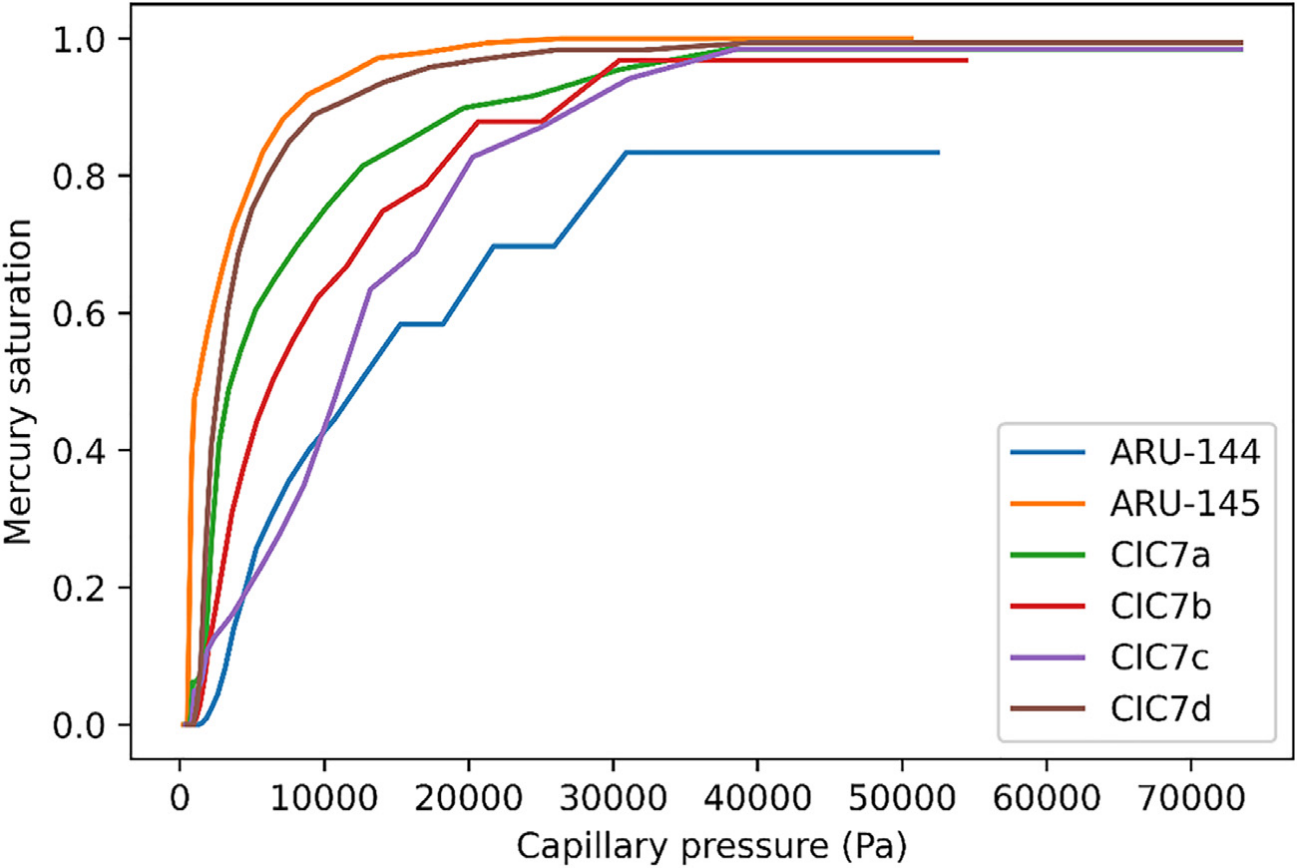
MICP (Mercury Intrusion Capillary Pressure) simulation curves of the scanned samples.

## Ethics Statements

Ethics statements are not required for the presented data. Our work did not involve human subjects, animal experiments, nor collect data from social media platforms.

## CRediT authorship contribution statement

**Mohamed Ali Elabd:** Methodology, Investigation, Formal analysis, Writing – original draft, Visualization. **Reneé González-Guzmán:** Conceptualization, Investigation, Resources, Writing – review & editing, Visualization, Funding acquisition. **Bodo Weber:** Writing – review & editing. **Corina Solís:** Writing – review & editing. **Rubén Bernard-Romero:** Writing – review & editing. **Fernando Velasco-Tapia:** Writing – review & editing. **Pedro Marín-Camacho:** Writing – review & editing.

## Declaration of Competing Interest

The authors declare that they have no known competing financial interests or personal relationships that could have appeared to influence the work reported in this paper.

## Data Availability

Two-D CT images of the host rock and layers of a sinter mound from Los Geysers (northern TMVB) (Original data) (Mendeley Data).
